# In Vivo Dual-Modal Photoacoustic and Ultrasound Imaging of Sentinel Lymph Nodes Using a Solid-State Dye Laser System

**DOI:** 10.3390/s20133714

**Published:** 2020-07-02

**Authors:** Moongyu Han, Wonseok Choi, Joongho Ahn, Hanyoung Ryu, Youngseok Seo, Chulhong Kim

**Affiliations:** 1 Department of Electrical Engineering, Creative IT Engineering and Mechanical Engineering, Pohang University of Science and Technology (POSTECH), Pohang 37673, Korea; dalgyu0820@postech.ac.kr (M.H.); zoids1215@postech.ac.kr (W.C.); joongho.ahn@postech.ac.kr (J.A.); 2 R&D Center, Wontech Co. Ltd., Daejeon 34028, Korea; hyryu@wtlaser.com (H.R.); physys@wtlaser.com (Y.S.)

**Keywords:** photoacoustic imaging, sentinel lymph node biopsy, solid state dye, methylene blue

## Abstract

Photoacoustic imaging (PAI) is being actively investigated as a non-invasive and non-radioactive imaging technique for sentinel lymph node (SLN) biopsy. By taking advantage of optical and ultrasound imaging, PAI probes SLNs non-invasively with methylene blue (MB) in both live animals and breast cancer patients. However, these PAI systems have limitations for widespread use in clinics and commercial marketplaces because the lasers used by the PAI systems, e.g., tunable liquid dye laser systems and optical parametric oscillator (OPO) lasers, are bulky in size, not economical, and use risky flammable and toxic liquid dyes. To overcome these limitations, we are proposing a novel dual-modal photoacoustic and ultrasound imaging system based on a solid-state dye laser (SD-PAUSI), which is compact, convenient, and carries far less risk of flammability and toxicity. Using a solid-state dye handpiece that generates 650-nm wavelength, we successfully imaged the MB tube positioned deeply (~3.9 cm) in chicken breast tissue. The SLNs were also photoacoustically detected in the in vivo rats beneath a 2.2-cm-thick layer of chicken breast, which is deeper than the typical depth of SLNs in humans (1.2 ± 0.5 cm). Furthermore, we showed the multispectral capability of the PAI by switching the dye handpiece, in which the MB-dyed SLN was selectively highlighted from the surrounding vasculature. These results demonstrated the great potential of the SD-PAUSI as an easy but effective modality for SLN detection.

## 1. Introduction

Photoacoustic (PA) imaging (PAI) has received considerable attention recently as an imaging technique for biomedical applications. The basic principle of PAI is based on the PA effect, which is the energy transduction of light into ultrasound (US) [1]. When a pulsed laser beam irradiates a biological tissue, the initial acoustic pressure at the tissue is increased due to the thermo-elastic expansion. The generated acoustic waves propagate towards the US transducers to be detected and reconstructed into images [2]. The contrast in PAI is determined by the difference in optical absorption of different tissues, and the spatial resolution is derived from the acoustic properties of the PA signals [3]. Thus, the PAI combines the advantages of both optical and ultrasound imaging and can probe a variety of optical endogenous or exogenous absorbents with high contrast and high spatial resolution, even at depths exceeding the optical diffusion limit (~1 mm) of the conventional optical imaging [4]. Thanks to these advantages, PAI systems have been applied to various preclinical and clinical applications such as detecting tumors by measuring the hypoxia or the abnormality in the microvasculature distribution [5,6,7], assessing ulcer formation [8,9] or peripheral vascular disease [10,11], diagnosing inflammatory arthritis [12], and so on.

The superiority of PAI mainly comes from its ability to image intrinsic optical chromophores, such as hemoglobin and melanin, without any external contrast agent [13]. However, not all biological targets have enough optical absorption contrast; some targets are almost transparent in the visible or near-infrared (NIR) regime, thus may not be detected with PAI without using external contrast agents [3]. One of the most prominent examples of a transparent target is the sentinel lymph node (SLN), which is the first target for metastasis in cancer patients such as melanomas and breast cancers [14,15,16] and a predictor of prognosis of cancer. SLN biopsy is currently the gold standard to determine the presence of cancer. However, SLN is not visible by itself. Thus, a surgeon locates the SLN after staining with dyes (e.g., methylene blue (MB)) and dissects the SLN to check for the presence of cancer cells through histopathologic examination. Prior to the SLN dissection, medical imaging procedures such as lymphoscintigraphy can be performed to non-invasively locate the SLN. The traditional SLN imaging methods using radioactive isotope materials (e.g., Technetium-99m) have various limitations, such as ionizing radiation, high cost, and poor spatial resolution [17,18]. Thus, contrast-enhanced PAI has been proposed as a promising non-invasive and non-radioactive imaging tool for detecting sentinel lymph nodes [19,20]. The PA SLN imaging locates SLNs noninvasively with various non-radioactive exogenous contrast agents such as nanoparticles [21,22,23,24,25], indocyanine green [26], and MB [27,28,29]. Most of the PAI systems for imaging SLN used liquid dye laser or optical parametric oscillator (OPO) laser as a light source. Both liquid dye laser and OPO laser have high power (tens of millijoules(mJ)) that enables deep-tissue imaging (~50 mm) and tunable laser wavelength, but they are not readily suitable for clinical deployment because of their high cost, bulky size, flammable and toxic solvents, and risks of skin and eye damage from lasers. Laser diodes (LD) and laser emitting diodes (LED) were recently presented as an illumination source as well [30,31,32,33]. LD and LED have high pulse repetition rates (in kHz range), low cost, and compact sizes, but the pulse energies are very low (hundreds of μJ to few mJ). To compensate for it, tens to hundreds of frames are averaged to enhance the signal-to-noise ratio (SNR). Also, the wavelength is not tunable but must be switched by altering the LD or LED.

In this paper, we demonstrate a dual-modal photoacoustic and ultrasound imaging system based on a solid-state dye laser (SD-PAUSI). We used a solid-state dye laser based on a polymeric dye, which was compact enough to be located in the laser handpiece and, thus, was very convenient, safe, and easy to use. The dye effectively changed the output wavelength of the laser to 650 nm, which is near the optical absorption peak wavelength of MB (656 and 667 nm) [34,35,36,37]. In addition, the laser beam was delivered through an articulated arm, which made the system more suitable for the clinical environment and was more durable than the fiber bundle. The solid-state laser is clinically approved and is currently being used in dermatology for tattoo and pigment removal. Thus, we believe the solid-state laser could be advantageous for regulatory approval to perform diagnostic PA imaging. We successfully imaged MB tubes positioned in deep biological tissues (~3.9 cm depth) in the in vitro experiment. We also conducted non-invasive in vivo imaging of the SLN in a rat injected with MB dye and observed the PA amplitude change for 15 min in the SLN after injection. In addition, we placed a 2.2-cm layer of chicken breast tissue anterior to the rat skin surface to evaluate the SLN imaging capability in deep tissues. We acquired multispectral images of an SLN at 532-, 650-, and 1064-nm wavelengths by manually switching the laser handpiece to show optically differentiated structures such as blood vessels, MB-dyed lymph vessels, and MB-dyed SLN [38]. These results confirmed the capability of the SD-PAUSI system as a potential imaging tool for PA SLN imaging.

## 2. Materials and Methods

### 2.1. Dual-Modal Photoacoustic and Ultrasound Imaging System with a Solid-State Dye Laser (SD-PAUSI) 

**Figure 1:**
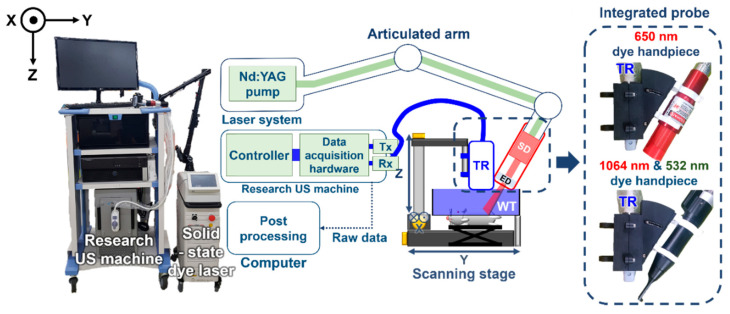
Photograph and schematic of dual-modal photoacoustic and ultrasound imaging system with a solid-state dye laser (SD-PAUSI). US, ultrasound; Tx, transmit; Rx, receive; TR, transducer; WT, water tank; ED, engineered diffuser; SD, solid-state dye.

**Figure 2:**
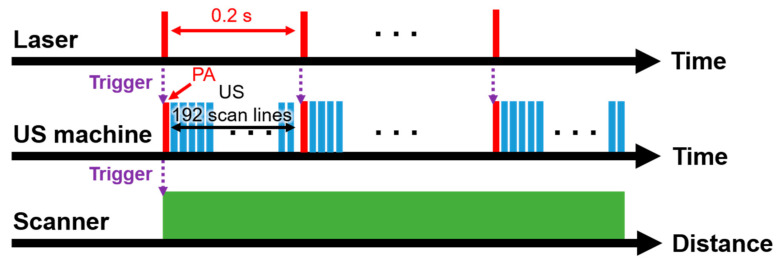
Data acquisition sequence of the dual-modal photoacoustic and ultrasound imaging system with a solid-state dye laser (SD-PAUSI). PA, photoacoustic; US, ultrasound.

The schematic and the photograph of the SD-PAUSI system are shown in Figure 1. The SD-PAUSI system integrates a solid-state polymeric dye laser (COSJET ATR, Wontech, Daejeon, Korea) and a research US imaging system (Vantage 256, Verasonics, Kirkland, WA, USA). The size of the laser system was 583 mm (X) × 288 mm (Y) × 847 mm (Z) and all components such as neodymium-doped yttrium aluminum garnet (Nd:YAG) pump laser or air cooling system were integrated in a single body. Maximum pulse energies at each wavelength were 1300 mJ, 500 mJ, and 150 mJ at 1064 nm, 532 nm, and 650 nm, respectively. The efficiency of the 650-nm solid-state dye was 40%, and both the stability and long-term drift were less than 10%. The lifetime of the 650-nm dye was 20 k–50 k shots. The 532-nm laser pulses were generated by a Q-switched Nd:YAG laser with pulse durations of 5–40 ns and a repetition rate of up to 10 Hz. The generated laser beam was delivered through an articulated arm to reach a 650-nm solid-state dye cell located in the laser handpiece which changed the wavelength from 532 nm to 650 nm. A 192-elements linear array US probe (GE9LD, General Electric Healthcare, Chicago, IL, USA) with a center frequency of 5.3 MHz was physically integrated with the dye handpiece to form a single integrated probe. The integrated probe was designed to flexibly adjust the laser illumination angle to align with the US imaging plane. The photographs of the integrated probes with 650-, 1064-, and 532-nm dye handpiece are shown in the dotted box in Figure 1. By switching the dye handpiece, 1064-nm or 532-nm laser beams from the Nd:YAG laser can be used. A water tank sealed with a thin transparent membrane provided acoustic coupling as well as space for the laser to shine directly onto the surface of the imaging plane. For volumetric imaging of the target, the imaging probe was moved across the target with a motorized scanning stage. The scanning stage moved continuously in the *y*-direction, and the US imaging system received PA/US raw data (*x-z* plane) for each laser pulse. Upon reception of each laser trigger pulse, PA raw data were received by all 192 US transducer elements, followed by US wave transmission and reception sequence corresponding to 192 scan lines to form the image. US data were acquired between every PA frame (Figure 2, Supplementary Video S1). The received PA/US raw data were processed and displayed in real time using pixel-based delay-and-sum beamforming while saving the PA/US beamformed image data for further processing. The saved beamformed data were exported to a personal computer to perform offline post-processing such as signal-to-noise ratio (SNR) calculation, maximum amplitude projection (MAP), or pixel intensity analysis.

### 2.2. In Vitro PA Imaging in Deep Tissues

**Figure 3:**
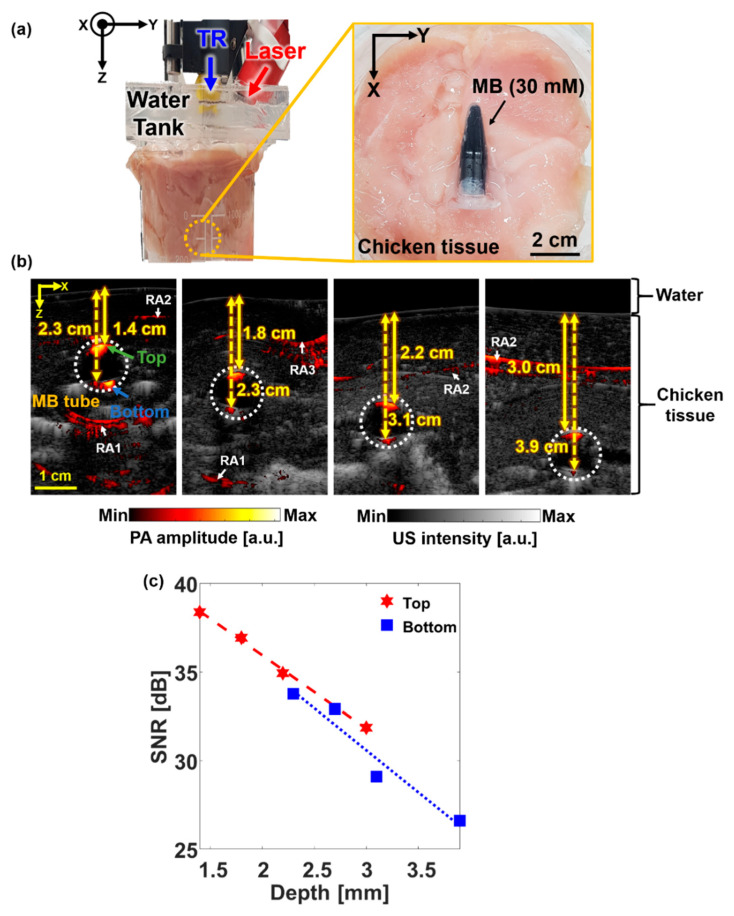
In vitro PA imaging of the MB tube at different depths. (**a**) Photographs of the experimental setup and the MB containing the tube positioned in chicken tissue. (**b**) Overlaid B-mode PA/US images at different depths. (**c**) SNRs of the PA images of 30-mM MB-filled tube at various depths from the tissue surface at 650-nm wavelength. TR, transducer; MB, methylene blue; SNR, signal-to-noise ratio; PA, photoacoustic; US, ultrasound; RA, reflection artifact; and a.u., arbitrary unit.

We investigated the deep-tissue imaging capability of SD-PAUSI by imaging MB in the in vitro experiments (Figure 3a). We imaged a transparent plastic tube filled with MB. The concentration of the MB was 30 mM (10 mg/mL), which is typically used in clinic for SLN biopsy [27,39,40]. A beaker was used to stack the chicken tissues. To prevent the laser or acoustic signals from getting reflected from the bottom surface of the beaker, an ~5-cm-thick expanded polystyrene foam was placed on the bottom surface before stacking the chicken. The MB tube was placed between chicken tissues with its long axis aligned perpendicular to the US imaging plane (x-z plane). A water tank with the bottom surface covered by the transparent plastic wrap was positioned over the beaker to couple the chicken tissue pile with the US probe. The laser handpiece was placed diagonally to the US probe using an integrated holder where the angle was adjusted to illuminate the surface of the imaging plane directly. The laser fluence was ~3.8 mJ/cm^2^ at 650 nm (pulse energy: 24 mJ, beam area: 6.4 cm^2^), which was well below the American National Standards Institute (ANSI) safety limit (20 mJ/cm^2^) [41]. To increase the imaging depth, we stacked multiple layers of chicken breast tissue until the PA signal from the target was not distinguishable from the background. All the acquired images were exported offline to calculate the SNR with respect to the imaging depth. To improve the SNRs, we averaged 50 PA image frames at each depth. 

### 2.3. In Vivo Rat SLN Imaging

All experiments were conducted in accordance with the laboratory animal use protocols approved by the Institutional Animal Care and Use Committee of Pohang University of Science and Technology. To obtain 3D volumetric images of the rat, the integrated probe mounted on the linear scanning stage acquired multiple cross-sectional (*x-z* plane) B-mode PA/US images while moving along the elevational direction (*y*-axis) (Figure 3a). The scanning range dimensions were 49.5 mm (lateral, *x*-axis) × 60 mm (elevational, *y*-axis) with the step sizes of 0.23 mm (*x*-axis, US probe pitch length) and 0.6 mm (*y*-axis), respectively. Laser pulse repetition frequency was 5 Hz, and the laser fluence was ~2.2 mJ/cm^2^ (pulse energy: 23 mJ, beam area: 10.5 cm^2^) on the skin. No frame averaging was applied in the in vivo studies. Three Sprague Dawley rats (~200 g) were initially anesthetized using vaporized isoflurane (1 L/min of oxygen and 0.75% of isoflurane) anesthetic system (VIP300, Midmark, Dayton, OH, USA) and the hair from the left axillary area of the rat was removed. A control PA image was obtained prior to the injection of MB (30 mM, 0.1 mL) and the MB was later injected intradermally into the left forefoot. For observing the change in the PA signal of the MB-stained SLN over time, PA images were acquired at intervals of 5 min up to 15 min. To investigate the deep SLN imaging capability of the SD-PAUSI system, we placed a layer of chicken tissue (~2.2 cm thick) over the rat and obtained PA images before and after MB injection. We also demonstrated multispectral PAI by manually changing the laser handpiece to utilize 1064- and 532-nm wavelengths. For analyzing the spectral trend, we normalized the PA signals of each wavelength with the measured average power (1064 nm, ~5.9 mJ/cm^2^; 650 nm, ~2.2 mJ/cm^2^; and 532 nm, ~2.8 mJ/cm^2^), and extracted the mean PA amplitudes from various body parts such as blood vessel, MB-dyed lymph vessel, and SLN. 

## 3. Results

### 3.1. In Vitro PA Imaging in Deep Tissues

Figure 3b shows the B-mode PA/US images of the MB tube inside the chicken tissues at different depths, where white, dashed circles indicate the cross-section of the MB tube. The US image shows the tube structure, and the PA image shows MB in the tube. Green and blue arrows point to the top and bottom of the MB tube, respectively. Multiple layers of 5-mm-thick chicken tissue were stacked one above the other to increase the imaging depth. The maximum detectable depth was 3.9 cm when the laser fluence was 3.8 mJ/cm^2^. The PA signal inside the tube was not apparent because it consisted of much lower frequency components than the US transducer bandwidth. PA signals appearing at other regions were comprised of reflection artifacts [42] and background PA signals from blood vessels in chicken tissue. The reflection artifacts were caused by the acoustic reflectors such as plastic tube (RA1), plastic wrap (RA2), and air gap (RA3), appearing at about twice the depth of each reflector. The rest of the background signals were the PA signals from the blood vessels in the chicken tissue. We also calculated the SNRs at different depths (Figure 3c), where SNR was the average of PA signals obtained either from the top or bottom of the tube divided by the standard deviation of the background signals at the same depths. The red stars and blue squares in Figure 3c represent the SNRs at the top and bottom of the tube, respectively. Both SNRs showed linear degradation along the depth with almost a similar slope. We calculated the effective optical effective attenuation coefficient $\left(\mu_{eff}\right)$ from the slope of the fitted line of SNR versus depth. The $\mu_{eff}$ values obtained from the top and bottom of the tube were 0.48 cm^−1^ and 0.55 cm^−1^, respectively. Both results were well within the previously reported result of 0.4 cm^−1^–1 cm^−1^ at 650 nm [43]. While chicken breast tissue has similar $\mu_{eff}$ with human breast (1.1 cm^−1^ [44]), the imaging depth in an actual live tissue could be somewhat lower than the depth measured in this experiment because of lack of blood.

### 3.2. In Vivo PA SLN Imaging Experiments 

**Figure 4:**
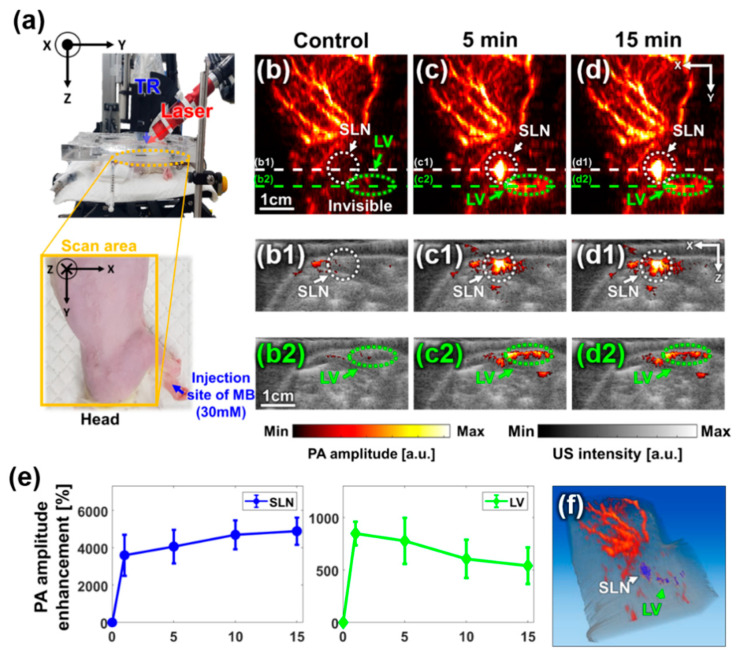
In vivo PA images of the rat sentinel lymph node over time. (**a**) Photographs of the experimental setup for in vivo rat imaging and the region of interest. (**b**–**d**) In vivo PA maximum amplitude projection (MAP) images of the rat over time. (**b1**–**d1**) Cross-sectional overlaid B-mode PA/US images cut along the white, dashed lines in Figures (**b**–**d**), respectively. (**b2**–**d2**) Cross-sectional overlaid B-mode PA/US images cut along green, dashed lines in Figures (**b**–**d**), respectively. (**e**) PA amplitude enhancement of the SLN and LV over time after injecting MB. (**f**) Snapshot of volume-rendered PA/US image (Supplementary Video S2). TR, transducer; MB, methylene blue; PA, photoacoustic; US, ultrasound; SLN, sentinel lymph node; LN, lymph node; and LV, lymph vessel, and a.u., arbitrary unit.

The experimental setup for in vivo SLN imaging is shown Figure 4a. The left side of the rat, which is marked with a yellow box, was scanned after removing the hair. The control PA image was acquired prior to MB injection, and the MB was injected into the left forefoot by using a 31-gauge insulin syringe. The PA images were acquired at 1 min, 5 min, 10 min, and 15 min post-injection to analyze the signal change over time (Figure 4b–d). White, dashed circles in the PA MAP images (Figure 4b–d) show the location of the SLN. In the control image, only the vascular structure is visible with no signal from SLN (Figure 4b). However, after injecting MB, the SLN is clearly visible with the strong PA amplitude (Figure 4c). The corresponding cross-sectional B-mode PA/US images, cut along the white, dashed lines in Figure 4b–d, are shown in Figure 4(b1–d1), respectively. Both the MAP images and the B-mode PA/US images show strong PA signals for the SLN. The lymphatic vessel (LV), through which MB travels to the SLN, is visualized both in the PA MAP image (green, dashed circles; Figure 4b–d) and the corresponding B-mode PA/US image slices (green, dashed circle; Figure 4(b2–d2)). We further quantified the enhancement in PA amplitudes of the SLN and LV with respect to time (Figure 4e). The enhancement was calculated as the ratio of the averaged PA signals in each region of interest (ROI) before and after MB injection. As MB travels through the LV and accumulates in the SLN, the PA signal in the SLN increased gradually while the signal in the LV decreased gradually soon after the injection. The PA signal at the SLN was enhanced >35 folds at 1 min post-injection and was further enhanced by ~50 folds at 15 min post-injection. In the case of LV, the peak signal enhancement was about nine folds at 1 min post-injection and then decreases gradually. Figure 4f shows the volume-rendered image of the overlaid PA/US images, where the SLN and LV are further highlighted with blue color, which is obtained by subtracting the control image from the image at 15 min post-injection [45] (Supplementary Video S2). The US image depicts the overall structural information of the rat body while the PA image highlights specific structures, including the vasculature, SLN, and LV. 

**Figure 5:**
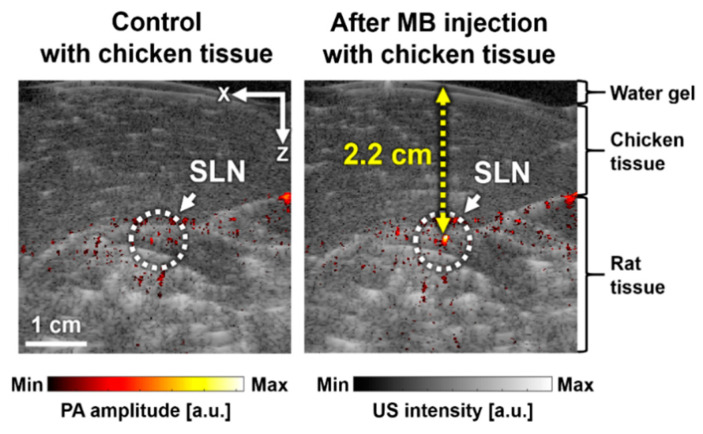
In vivo cross-sectional B-mode PA/US images with chicken tissue before (**a**) and after (**b**) MB injection. SLN, sentinel lymph node; MB, methylene blue; and a.u., arbitrary unit.

To show the deep-tissue imaging capability, we imaged the SLN dyed with MB after stacking a layer of chicken tissue on top of the rat in vivo (Figure 5). The thickness of the chicken tissue was ~2.2 cm, which is deeper than the typical depth of SLN in humans (approximately 1.2 ± 0.5 cm) [40]. The cross-sectional B-mode PA/US images of the rat pre- and post-injection of MB are shown in Figure 4a,b, respectively. The SLN positioned deeply (~2.2 cm) was detected with a PA signal enhancement of 170 ± 25%. This result showed the feasibility of our SD-PAUSI system to image the SLN in vivo in humans.

### 3.3. In Vivo Multispectral PA SLN Imaging 

**Figure 6:**
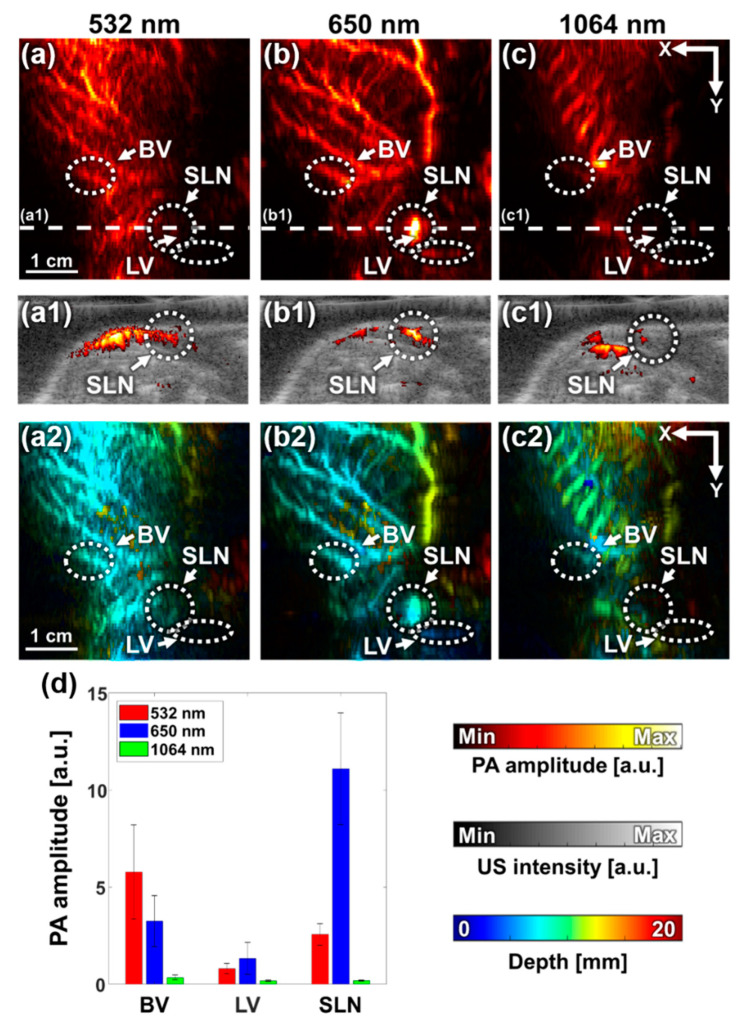
In vivo multispectral PA imaging of rat sentinel lymph node. (**a–c**) PA MAP images obtained with 532-, 650-, and 1064-nm wavelengths, respectively. (**a1–c1**) Cross-sectional B-mode PA/US images at white, dashed lines in Figures (**a–c**), respectively. (**a2–c2**) Depth-encoded PA MAP images. (**d**) Quantified PA amplitudes at BV, LV, and SLN with different wavelengths. PA, photoacoustic; BV, blood vessel, LV, lymph vessel; SLN, sentinel lymph node; and a.u., arbitrary unit.

We performed multispectral PA imaging with optical wavelengths of 532, 650, and 1064 nm by manually switching the 650-nm and 532/1064-nm handpieces. Figure 6a–c shows the PA MAP images acquired at 532-, 650-, and 1064-nm wavelengths, respectively. The PA MAP image at 532 nm (Figure 6a) mostly shows the vascular structure, while the image at 650 nm additionally depicted SLN and LV dyed with MB. These results accurately described the spectral tendencies of hemoglobin and MB, which have a strong absorption peak near 532 nm and 650 nm, respectively. The PA MAP image at 1064 nm shows the rib cage and the surrounding vasculature. Figure 6(a1–c1) shows the B-mode PA/US images sliced at the corresponding white, dashed lines in Figure 6a–c. The B-mode PA/US images clearly showed that the SLN was selectively detected at 650 nm. The depth-encoded PA MAP images of the three wavelengths are shown in Figure 6(a2–c2), where the depth was calculated with respect to the shallowest location of the skin. The image obtained at 532 nm shows blood vessels in relatively shallow depths (around 0–1 cm), while 650 nm shows more vessels in deeper depths (up to 1.3 cm). Thus, the thick vessel depicted with yellow color in the vertical direction in Figure 6(b2) is better visualized at 650 nm than in 532 nm.

To analyze the PA signals quantitatively, we compared the PA amplitudes of BV, LV, and SLN at each wavelength after normalizing them with the corresponding average laser pulse energy (Figure 6d). The PA signals were calculated as the mean pixel values in the white, dashed circles in Figure 6a–c, and the error bars indicate the standard deviations of the individual pixel values in each ROI. The PA signal from BV tended to decrease from 532 to 1064 nm, which resembled the optical absorption spectrum of hemoglobin. Similarly, PA signals from SLN and LV followed the optical absorption tendency of MB, which has a peak around 650 nm.

## 4. Discussion

We developed a dual-modal PA/US SLN imaging system (SD-PAUSI) by combining a solid-state dye laser and an US imaging system. This system overcame the limitations of the existing PAI systems, which use liquid dye lasers or OPO lasers. The SD-PAUSI was able to detect the PA signal of MB tube at a depth of 3.9 cm in vitro, and successfully imaged the rat SLN underneath the 2.2-cm-thick chicken tissue layer post MB injection. The SD-PAUSI presented here has many advantages over conventional techniques in terms of size, scalability of the application, and clinical translatability. The capability of producing high power combined with the small size of the entire laser body and the handpiece makes it most suitable for deployment into the clinical environments. We showed the clinical feasibility of the system by imaging the SLN at 2.2 cm depth, which is beyond the typical depth of SLNs in humans. In addition, the switchable solid-state dye would potentially extend its applications, e.g., 532 nm or 650 nm for vascular imaging, 650 nm for MB-based SLN imaging, and 1064 nm for deep-tissue imaging. Since the solid-state dye laser has already been approved for the therapeutic applications in dermatology, which uses high laser fluence, we expect our system to be translated easily for diagnostic use where much less fluence is used. 

The SD-PAUSI system could be further improved in several aspects as described below. Currently, the ultrasound probe was fixed vertically, and the laser was irradiated obliquely onto the surface of the skin (Figure 1). Thus, an offset with a transparent water tank was necessary for the coincidental alignment of the PA/US imaging plane with the laser illumination. However, the size is not yet suitable for handheld operation and it is cumbersome to use the water tank in clinic. In the future, if the laser is integrated with the ultrasound probe to co-axially illuminate the target by incorporating optical/acoustic beam combiner, the probe will become much more feasible for handheld operation. In addition, we are planning to reduce the reflection artifact from the probe surface using white acoustic lens having much less light absorption. After the probe size is optimized, we will aim to develop a 3D imaging probe by combining it with a handheld motorized scanner [46]. For multispectral PA imaging, the laser wavelength was changed manually by switching the handpiece. Thus, it was difficult to image the anatomy precisely at the same location for each wavelength [38]. If the solid dye component can be switched automatically in a single handpiece or inside the laser body, it would be possible to minimize motion artifacts during wavelength switching and perform accurate spectral unmixing of multi-wavelength PA images. In addition, we did not apply a specific method to correct motion artifacts typically caused by breathing. If we use autocorrelation between consecutive frames, it is possible to improve image quality by compensating for the motion. Further, because the elevational focus of US transducer was fixed at 28 mm depth, thus the width of the acoustic beam away from the focal depth would be increased. To overcome it, synthetic aperture focusing technique might be able to enhance the resolution in the out-of-focus regions [47].

## 5. Conclusions

We developed a compact, easy-to-use, dual-modal SD-PAUSI system for SLN imaging. We successfully detected in vitro MB (30 mM) in a biological tissue up to a depth of 3.9 cm. In the in vivo imaging of the rat SLN, the PA images using our system clearly delineated the SLN and PA amplitude change over time after intradermal injection of MB. The detectability inside deep tissue was also further confirmed by stacking chicken breast tissue (~2.2 cm) over the SLN. In addition, we also showed the multispectral PA imaging capability of the system by imaging at 532, 650, and 1064 nm wavelengths that selectively emphasized vasculature, MB-dyed SLN and LV, and rib cage, respectively. These results highlight the potential of the solid-state polymeric dye laser for PA imaging of SLNs and multi-structural deep-tissue biomedical imaging. We believe, with these advantages over conventional systems, our system would be easily translated to clinical use in the near future.

## Supplementary Materials

The following are available online at https://www.mdpi.com/1424-8220/20/13/3714/s1, Video S1: Real-time B-mode PA/US image of the in vivo rat. Video S2: Volume-rendered PA/US image of the in vivo rat at 15 min after MB injection.
